# Isolation and Identification of Fourteen Microsatellite Markers in *Clivia miniata* and *Clivia nobilis* (Amaryllidaceae)

**DOI:** 10.3390/ijms13089609

**Published:** 2012-08-02

**Authors:** Wei Gao, He Zhao, Ying Liu, Ming-Rui Li, Eliyas Nurmamat, Lin-Feng Li, Yue-Ying Ren, Hong-Xing Xiao

**Affiliations:** 1 College of Traditional Chinese Medicinal Material, Jilin Agricultural University, Changchun 130118, China; E-Mail: sgaov@yahoo.com.cn; 2 College of Biological Science and Technology, Changchun University, Changchun 130012, China; 3 Key Laboratory of Molecular Epigenetics of Ministry of Education, Northeast Normal University, Changchun 130024, China; E-Mails: zhaoh036@nenu.edu.cn (H.Z.); liuy248@nenu.edu.cn (Y.L.); limr145@nenu.edu.cn (M.-R.L.); lilf241@nenu.edu.cn (L.-F.L.); 4 College of Chemistry and Life Sciences, Yili Normal University, Yili, 835000, China; E-Mail: nuemmt496@nenu.edu.cn

**Keywords:** *Clivia miniata*, *Clivia nobilis*, genetics diversity, microsatellite

## Abstract

*Clivia* is a genus of great horticultural importance and has been widely cultivated as ornamental plants in all over the world. In order to assess the phylogenetic relationships and genetic diversity of the wild *Clivia* species and cultivars, we isolated AC-enriched repeats using FIASCO from a single clone each of *C. miniata* Regel. and *Clivia nobilis* Lindl. Of the fourteen repeats, 10 were polymorphic and 4 were monomorphic. The polymorphic marker loci were characterized using 61 *Clivia* accessions. The number of alleles ranged from two to six, observed heterozygosity ranged from 0.04 to 1.00 and expected heterozygosity ranged from 0.04 to 0.83. These microsatellite marker loci provide tools for future studies of *Clivia* species and cultivars.

## 1. Introduction

The genus *Clivia* Lindl. belongs to the family Amaryllidaceae and includes six diploid species (2*n* = 2*x* = 22) primarily distributed in southern Africa [1–3]. *Clivia*, an evergreen genus with rhizomes, prefers well-drained, semi-shade and shaded habitats [4–6]. As one of the famous ornamentals with colorful flower and tremendous economic values, the species within *Clivia* have been cultivated in many parts of the world and lots of cultivars have been developed in the past decades [5,7]. To gain a better understanding of the phylogenetic relationships within the genus *Clivia*, several previous studies have constructed the phylogenies of *Clivia* using RAPD markers and DNA sequences [7–9]. The results of these studies provide us fundamental insight into the systematic relationships among the six congeneric species. Nevertheless, these wild *Clivia* species were usually used as genetic resources for the interspecific cross-breeding [5,10–12]. Therefore, the phylogenetic relationships and genetic erosion of these wild *Clivia* species and cultivars remain unclear due to the lack of reliable and efficient genetic and molecular markers. To obtain the genetic composition of the wild *Clivia* species and cultivars, we isolated and identified fourteen genomic microsatellites from the species *Clivia miniata* Regel. and *Clivia nobilis* Lindl. These microsatellite primers provide us a valuable resource to evaluate the phylogenetic relationships and genetic diversity of wild *Clivia* species and cultivars.

## 2. Results and Discussion

A total of 68 positive clones for *C. miniata* and 14 for *C. nobilis* were sequenced, of which 33 clones of them (26 from *C. miniata* and seven from *C. nobilis*) containing enough flanking regions (>30 base pair) were successfully designed primer. Eleven microsatellites from *C. miniata* (CM3, CM9, CM12, CM54, CM65, CM103, CM137, CM253, CM289, CM357 and CM425) and three from *C. nobilis* (CN68, CN89 and CN106) producing clear amplicons of the expected size were selected for the following experiment, and the other primer pairs which amplified multi-bands were abandoned in this study. The band size is scored against a 25 base pair DNA marker (Takara). Finally, four of these primer pairs were monomorphic (GenBank numbers: JQ782264, JQ782270, JQ782274 and JQ782277), and the other 10 polymorphic loci produced a total of thirty-five alleles, with an average of 3.5 alleles per locus. The number of alleles per locus (*N*_A_) was two to six, and the observed heterozygosity (*H*_O_) and expected heterozygosity (*H*_E_) varied from 0.04 to 1.00 and from 0.04 to 0.83, respectively (Table 1). No significant linkage disequilibrium was detected for any pair of loci.

## 3. Experimental Section

### 3.1. Isolation of Microsatellite Markers

The samples for this study were collected from fifty-one cultivars of *C. miniata*, eight wild individuals of *C. nobilis* and two hybrids between the two species. The microsatellite-enriched libraries were constructed following the procedure of Fast Isolation by AFLP of Sequences Containing repeats (FIASCO) [13]. In brief, total genomic DNA was extracted from a single individual each of *C. nobilis* and *C. miniata*. About 300 ng genomic DNA for each species was completely digested with *Mse*I separately (New England BioLabs, Beverly, MA, USA). Then, these digested products were ligated to the MseI adaptor pair (5′-TACTCAGGACTCAT-3′/5′-GACGATGAGTCCTGAG-3′) [14]. The diluted digestion-ligation mixture (1:10) was then amplified with *Mse*I-N primer (5′-GATGAGTCCTGAGTAAN-3′) using the following cycle conditions: 95 °C for 5 min, 20 cycles of 94 °C for 30 s, 53 °C for 1 min, 72 °C for 1 min, and a finally extension at 72 °C for 5 min.

For enrichment, these PCR products were denatured and hybridized with biotin-labeled (AC)_15_ probes, and then these DNA fragments were captured by streptavidin-coated magnetic beads (Promega, Madison, WI, USA). These enrichment products were purified with a Gel Extraction Kit (Takara, Liaoning, China) and ligated into the pMD18 vector (Takara) and transformed into *E. coli* strain JM109 (Takara). The positive clones were identified by PCR amplification using (AC)_10_ and M_13+_/M_13−_ primers, respectively. If one of the primer combinations was successfully amplified, it suggested that the amplicons contained microsatellite motifs. The positive clones for *C. miniata* and *C. nobilis* were sequenced on an ABI 3730 DNA analyzer (Applied Biosystems, Foster City, CA, USA) using M13F as sequencing primer. Primers were designed according to Li *et al*. [15]: 1. the length of primer was 18–25 bp; 2. annealing temperature was 55–65 °C; 3. GC content of primer was 40–60%.

### 3.2. Detection of Polymorphism and Data Analysis

Primer pairs were used to amplify DNA of 61 individuals. PCR amplifications were performed in a total volume of 20 μL containing the following components: 50 ng of DNA template, 0.2 μM of each dNTP (10 mM), 0.5 pmol of each primer, 1× PCR buffer (containing 2.5 mM Mg^2+^) and 1 U of high-fidelity *Taq* polymerase (Takara). The PCR reactions were performed on an ABI 2720 thermocycler: an initial denaturation of 5 min at 95 °C, 35 cycles of 30 s at 94 °C, annealing temperature (Ta) for 30 s (Ta for each primer pairs were listed in Table 2), 30 s at 72 °C, and a final extension of 72 °C for 8 min. These amplification products were resolved on 6% polyacrylamide denaturing gel electrophoresis and visualized using silver staining. To discriminate the real alleles from *Taq* polymerase errors and nonspecific amplification products, only clear bands with expected size were considered and the rare alleles (only occured once) were removed from the dataset.

The preliminary population genetic analyses for these microsatellite loci were performed using the software GENEPOP with the default settings and assumptions [16], including the number of alleles per locus (*N*_A_), observed heterozygosity (*H*_O_), expected heterozygosity (*H*_E_) and linkage disequilibrium (LD).

## 4. Conclusions

In this study, fourteen microsatellite markers were isolated and identified from the species *C. miniata* and *C. nobilis*. Ten polymorphic microsatellite markers may be useful in future studies of conservation biology and population genetics of the wild *Clivia* species and cultivars.

## Figures and Tables

**Table 1:** Results of initial primer screening in *Clivia miniata*, *Clivia nobilis* and hybrids of the two species. Parameters shown for each pair of primers are the number of the samples (*N*), number of alleles (*N*_A_), observed heterozygosity (*H*_O_) and expected heterozygosity (*H*_E_).

	*Clivia miniata* (*N* = 51)			Hybrid (*N* = 2)			*Clivia nobilis* (*N* = 8)		
			
Locus	*N*_A_	*H*_E_	*H*_O_	*N*_A_	*H*_E_	*H*_O_	*N*_A_	*H*_E_	*H*_O_
CM9	3	0.17	0.18	1	0.00	0.00	1	0.00	0.00
CM12	4	0.22	0.18	3	0.83	0.50	3	0.49	0.38
CM54	1	0.00	0.00	1	0.00	0.00	2	0.22	0.00
CM65	2	0.09	0.10	1	0.00	0.00	1	0.00	0.00
CM103	4	0.48	0.53	2	0.50	0.50	1	0.00	0.00
CM137	2	0.04	0.04	1	0.00	0.00	1	0.00	0.00
CM289	3	0.44	0.65	2	0.50	0.50	4	0.42	0.38
CM357	5	0.56	0.76	3	0.83	0.83	2	0.20	0.00
CN68	4	0.23	0.16	1	0.00	0.00	3	0.30	0.00
CN106	2	0.50	1.00	2	0.67	1.00	2	0.47	0.88

**Table 2:** Characteristics of ten microsatellite markers based on 61 *Clivia* accessions. For each locus, the names, primer sequences [forward (F) and reverse(R)], repeat motif, size of the cloned allele, annealing temperature, number of alleles and GenBank accession number are shown.

Locus	Primer sequences (5′–3′)	Repeat	Size (bp)	*T*_a_	*N*_A_	GenBank
CM9	F: TTACCTCCTCCAACTCACAG R: ATGAAAACGGCCCGAATTAC	(TG)_9_	251	48	3	JQ782265
CM12	F: CAACCTCAAGCTCAGTCTCA R: AGGTGCAGGATGATAATGGT	(AC)_6_T(AC)_2_	230	47	4	JQ782266
CM54	F: GAGCAACTCAGGAAGGGATG R: CCGCTAAGGACTTTACGCAC	(GT)_16_	194	46	2	JQ782267
CM65	F: AGGGTGAGAGTGTAGGTGTG R: CTTAGGCATGTATAGTGGCC	(GT)_4_T(TG)_4_	190	48	2	JQ782268
CM103	F: ACCCCTTACACAGACTACCA R: GATGGGTAGAAGGTGGTTGT	(AC)_15_G(CA)_21_	313	47	4	JQ782271
CM137	F: TTCTTGCCGACAATCCCGTA R: TGATGGTCGCAAAATTCACG	(AC)_6_	288	47	2	JQ782273
CM289	F: GGATATTATGTTGCCAAGCC R: AAAGTTGAATCGCGGTCCCA	(TG)_11_	207	58	5	JQ782275
CM357	F: GCGAATGATAATCGTGAACC R: TCCTCCTTTTCTCACACTTC	(GT)_16_	160	46	6	JQ782276
CN68	F: TTCTGACTTACACCTACACA R: ATTATGCACTTGGATCTAGG	(AC)_17_	167	47	5	JQ782269
CN106	F: AACAGCAGGAAATTAGGGAG R: TCACAAGCATACACACACAC	(TG)_16_	195	48	2	JQ782272
